# Comparative safety of cholinesterase inhibitors and memantine for dementia: a protocol for a network meta-analysis of randomized controlled trials

**DOI:** 10.1186/s13643-025-02961-6

**Published:** 2025-11-04

**Authors:** Moaz Elsayed Abouelmagd, Nereen A. Almosilhy, Hamdy A. Makhlouf, Amr K. Hassan, Ahmed S. A. Osman, Mahmoud Diaa Hindawi, Ahmed Elshahat, Asmaa Zakria Alnajjar, Mohamed M. M. Mustafa, Omar Khaled Abdelsalam, Abdelrahman Mady, Ahmed Shaheen, Matthew J. Barrett, Ahmed Negida

**Affiliations:** 1 Kasr-Alainy Faculty of Medicine, Cairo University, Cairo, Egypt; 2 Department of Pharmacology and Toxicology, Faculty of Pharmacy, Tanta University, Tanta, Egypt; 3 Faculty of Medicine, Minia University, Minia, Egypt; 4 School of Medicine, University of California, Irvine, CA USA; 5 Faculty of Medicine, Al-Azhar University, Cairo, Egypt; 6 Faculty of Medicine, Al-Azhar University, Gaza, Palestine; 7 Faculty of Pharmacy, Egyptian Russian University, Badr City, Egypt; 8 Faculty of Medicine, New Mansoura University, New Mansoura, Egypt; 9 Alexandria Faculty of Medicine, Alexandria, Egypt; 10 Department of Neurology, Virginia Commonwealth University, Richmond, VA USA; 11 Medical Research Group of Egypt, Negida Academy, Arlington, MA USA; 12 BrainLat Institute, School of Psychology, Adolfo Ibáñez University, Santiago, Chile

**Keywords:** Dementia, Cholinesterase inhibitors, Memantine, Adverse events, Network meta-analysis, Protocol

## Abstract

**Background:**

Dementia is a growing public health concern, affecting over 55 million people worldwide, with Alzheimer’s disease (AD) being the most prevalent cause. Cholinesterase inhibitors (ChEIs) and memantine remain the mainstay pharmacological treatment for AD and other dementias, despite their modest benefits and potential adverse effects. The safety profiles of these medications, particularly at different doses and formulations, remain inadequately explored, necessitating a comprehensive evaluation.

**Methods:**

This systematic review and network meta-analysis (NMA) will assess the safety of ChEIs (donepezil, galantamine, rivastigmine) and memantine in dementia treatment. We will include randomized controlled trials (RCTs) with ≥ 3 months of follow-up, evaluating adverse events (AEs), serious adverse events (SAEs), and treatment discontinuation rates. A comprehensive literature search will be conducted in PubMed, Scopus, Web of Science, and Cochrane Library, with additional searches in Google Scholar and reference lists of included studies.

Data extraction will follow a standardized approach, and study quality will be assessed using the Cochrane risk-of-bias tool-2. A Frequentist or Bayesian NMA framework will be used to compare safety profiles, with heterogeneity assessed using the I^2^ test.

**Discussion:**

By addressing gaps in prior NMAs, this study aims to provide an in-depth evaluation of safety outcomes associated with different ChEI and memantine doses and formulations across various dementia types. The findings will support clinicians in making informed treatment decisions and guide future research and policy development for dementia management.

**Systematic review registration:**

PROSPERO (CRD42025642902).

**Supplementary Information:**

The online version contains supplementary material available at 10.1186/s13643-025-02961-6.

## Background

Dementia is a disease of aging, affecting 3% of individuals aged 65–70 and increasing to 24–33% by age 85, with over 55 million cases worldwide-60% of them in low- and middle-income countries-and nearly 10 million new cases annually [1–3]. Dementia is currently ranked as the seventh leading cause of death leading to irreversible deterioration of the brain’s nerve cells; impairing memory, decision-making, and daily activities [2, 4]. With dementia cases rising, over 11 million U.S. caregivers in 2022 faced emotional, mental, physical, and financial challenges [5–8]. In 2019, dementia costs reached $1.3 trillion globally, half borne by informal caregivers like family and friends [2]. Given the predicted rise in dementia prevalence, disability, and dependency, prevention and treatment have become global health priorities [9].

Alzheimer’s disease (AD) is the leading cause of dementia, responsible for 60–80% of cases, with U.S. cases anticipated to double from 6.7 million in 2023 [10–12]. Other causes include frontotemporal dementia (FTD), which, along with AD, is a key cause of early-onset dementia before age 65, accounting for 10% of cases. It is also present in approximately 3% of individuals aged 65 and older [3, 13]. In addition, vascular dementia (VaD), represents 5–10% of dementia cases, and dementia with Lewy bodies disease (DLB) which present in 5% of older dementia patients [10, 11, 14]. Hippocampal sclerosis (HS) causes dementia in 0.4% to 2% of cases [15]. However, over half of AD patients, especially those aged 85 and older, have mixed dementia with multiple underlying causes [11].

As a result of the limited treatment options, cholinesterase inhibitors (ChEIs) (Donepezil, Galantamine, Rivastigmine) and Memantine, a partial antagonist of N-methyl-D-aspartate receptor (NMDAR), remain the primary treatment line for AD and non-AD dementias [8]. Despite providing modest cognitive benefits, they have been shown to relieve symptoms, slow disease progression, and reduce mortality and caregiving burdens [16–19]. Thereby, approximately 50% of dementia patients are prescribed ChEIs as part of their treatment [20]. Donepezil (a long half-life reversible ChEI) and Rivastigmine (a short half-life pseudo-irreversible ChEI) are approved for mild to severe AD, while Galantamine (a short half-life reversible ChEI and presynaptic nicotinic receptor modulator) is approved for mild to moderate AD, and Memantine is indicated for moderate to severe AD [8, 21]. All demonstrated cognitive benefits in non-AD dementias such as DLB and Parkinson's disease dementia (PDD), while Donepezil and Memantine have also shown benefits for VaD and traumatic brain injury (TBI) dementia [8, 22–26]. Nevertheless, none are supported for FTD due to insufficient efficacy [27].

A previous meta-analysis by Huo et al. [28] concluded that both ChEIs and memantine significantly improved cognition, functional abilities, global deterioration, and behavioral symptoms of dementia. Although treatment with ChEIs was associated with increased healthcare costs (MD: 859, 95% CI [− 150 to 1847]). In contrast, memantine reduced both healthcare costs (MD: − 2283, 95% CI [− 7874 to 3309]) and societal costs (MD: − 6322, 95% CI [− 14,355 to 1711]) [28]. Of note, the economic impact of combination therapy was unclear due to the lack of sufficient research, compared to monotherapy [28]. Therefore, although dementia treatments are expensive, they highlight the critical need for cost-effective policies and decision-making, especially due to the growing healthcare demands and the limited resources of aging societies [29, 30].

Furthermore, ChEIs are generally well-tolerated and are commonly associated with commonly known cholinergic side effects, such as nausea, vomiting, diarrhea, bradycardia, and dizziness [31]. However, reports of ChEI-induced adverse events (AEs) have increased, with 70% classified as serious adverse events (SAEs) and up to 2.3% resulting in fatalities, raising debates about their long-term safety [32–34]. Population-based studies have linked ChEIs with higher rates of bradycardia, syncope, hip fractures, and pacemaker insertion in older dementia patients [35–37]. Discontinuation of ChEIs after long-term use in patients with moderate to severe AD is common in practice, with higher risks of cognitive decline and functional impairment, requiring a careful assessment of the patient's risks, costs, and benefits [38, 39]. The prolonged use of memantine has been associated with SAEs, including seizure-like episodes and higher treatment withdrawal due to AEs [40, 41]. Interestingly, a study by Knapp et al. [42] showed that continuation of donepezil in patients with moderate to severe AD for 52 weeks was more cost-effective than discontinuation, regardless of outcomes (e.g., cognitive or functional improvements) [42]. Thus, most economic evaluations generally recommend the long-term use of ChEIs (4–5 years) because it is cost-effective [43, 44].

### Rational

Research on the safety of ChEIs and memantine in the management of dementia remains limited. In 2018, Dou et al.’s NMA included 41 RCTs on AD dementia; revealing that Galantamine (24–32 mg daily) and donepezil (10 mg daily) showed the most cognitive benefits for mild to moderate AD, while the combination of memantine (20 mg) and donepezil (10 mg) was recommended for moderate to severe cases [45]. Veroniki et al. [46] conducted an NMA of 80 RCTs to compare the safety and efficacy of ChEIs and memantine in managing AD dementia. The analysis indicated that donepezil, both alone and in combination with memantine, improved cognitive outcomes, particularly in moderate-to-severe impairment; however, donepezil and oral rivastigmine were associated with the highest risk of AEs [46]. Veroniki et al. did not discriminate between different doses of the drugs and both studies assessed safety through the rate of overall adverse events only and found memantine to have the highest tolerability [46]. Guo et al. [47] evaluated the effectiveness and cost of donepezil and memantine. Based on 54 trials in English and Chinese, Guo and colleagues concluded that donepezil combined with memantine showed greater efficacy than monotherapy or placebo in cognition. Although they measured safety through two outcomes (discontinuation rates and adverse events), they had a limited scope by only concentrating on two drugs [47]. Moreover, a Cochrane NMA by Battle et al. [24] examined eight trials assessing the efficacy and safety of ChEIs in VaD. The analysis demonstrated that donepezil (5–10 mg) and galantamine provided slight, clinically insignificant cognitive benefits for VCI, with donepezil 10 mg showing the greatest benefit but with notable side effects, which may still be worth considering in the absence of alternative treatments [24]. Moreover, Battle et al. assessed safety through 3 outcomes (number of adverse events, SAEs, death), missing some of the most important safety outcomes [24].

Nonetheless, previous NMAs have demonstrated significant limitations in evaluating the safety of ChEIs and memantine for dementia. First of all, none of the earlier studies conducted an in-depth analysis of the safety outcomes and specific adverse events related to each drug, where it’s common to associate ChEIs with more GIT symptoms and memantine with more CNS and psychological symptoms. Additionally, some of the previous NMAs failed to evaluate the different dosages and formulations of the drugs, usually combining all of them, where Adverse events of these drugs and their efficacy are mostly dose-dependent, leading to potential imprecise and over-generalized results and statements on the drugs. An additional problem arises in evaluating different doses of ChEIs. A “dose-range” in the outcomes resulting from the trials’ design limits the clinical interpretation of the outcomes (e.g., Galantamine 24–32 mg). Furthermore, these studies have primarily concentrated on AD dementia, often excluding other dementia types, such as VaD and PDD.

Most of the analyses of previous NMAs focus on the efficacy of interventions rather than safety profiles, concentrating on one safety outcome (AEs) or two. At the same time, specific adverse events and serious or fatal adverse events are undervalued. Treatment discontinuation due to AEs has also been significantly underreported. Providing data on total, serious, and discontinuation rates associated with dementia interventions is specifically important for research and practice, where studies suggest that a large number of patients discontinue their medications while clinicians have to balance their decision between efficacy and adverse effects of these drugs [48, 49]. This is where we also aim to provide specific system-related adverse events as secondary outcomes to allow evidence-based personalized approach in treating dementia.

Addressing these gaps is crucial to ensure balanced evaluations, informed clinical decisions, and effective regulatory policies to support optimum care for patients with this devastating disease.

Therefore, the objectives of our systematic review and network meta-analysis are outlined as follows:

I. Identify all RCTs exploring the safety of ChEIs, including Donepezil, Galantamine, Rivastigmine, and memantine, across different doses and preparations in managing various types of AD and non-AD dementias, such as VaD and PDD.
II. Assess the safety of ChEIs and memantine in treating dementia with several comprehensive important outcomes at various doses and formulations.
III. Provide clinicians with detailed guidelines on expected specific adverse events for each drug for better decision-making.
IV. Support future trials to calculate adequate sample sizes accounting for discontinuation rates.

## Methods

Our study will be conducted in accordance with the recommendations of the Cochrane Handbook for systematic review and meta-analysis and PROSPERO. Our protocol has been registered on PROSPERO with the number CRD42025642902. See Supplementary Material for PRISMA-P checklist.

### Study selection and eligibility criteria

Search will be conducted for relevant keywords across the most famous databases, including PubMed, Scopus, Web of Science, and Cochrane Library. Additionally, searching references of included studies and a manual search of Google Scholar for relevant RCTs will be conducted. The following search strategy will be used and tailored for each database is:((acetylcholinesterase inhibitor*) OR (cholinesterase inhibitor*) OR (donepezil) OR (galantamine) OR (galanthamine) OR (rivastigmine) OR (memantine)) AND ((dementia) OR (alzheimer) OR (alzheimer’s)OR (vascular dementia) OR (lewy body dementia) OR (dementia with lewy body) OR (parkinson* disease dementia) OR (frontotemporal demetia)) AND (randomized OR randomised OR "controlled trial" OR RCT OR "clinical trial" OR "random allocation" OR "randomly assigned").

After that, the identified reports will be imported into the Rayyan online software for duplicate detection and deletion. Six authors will be responsible for double screening the remaining reports on Rayyan, blinded from each other, and the first author (M.E.A.) will be responsible for conflict resolution. Following the initial screening process, A similar process will be conducted for full-text screening with the same authors. Articles that could not be retrieved will be requested from the corresponding authors through email. We will report a PRISMA flow diagram detailing all steps of the selection process and causes for exclusion.

We will include randomized controlled trials (RCTs) evaluating the safety of cholinesterase inhibitors (donepezil, galantamine, rivastigmine) or memantine with ≥ 3 months follow-up in participants diagnosed with dementia of any subtype (AD, VaD, dementia with Lewy bodies DLB, Parkinson’s disease dementia [PDD], or frontotemporal dementia [FTD]) across all stages. 3 months was chosen as a time point to allow for delayed adverse events and a comprehensive evaluation of all possible adverse events [50]. Diagnostic criteria include validated frameworks such as the DSM-5 (APA 2013) or ICD-11. Studies describing "undifferentiated dementia" or lacking subtype specificity will be excluded. Rare dementia subtypes (e.g., CADASIL) will also be excluded a priori.

Interventions were all doses and formulations (oral or transdermal) of donepezil, galantamine, rivastigmine, or memantine (monotherapy or combination therapy), compared to placebo or other active comparators included in our study. Doses were categorized as follows:

- *Donepezil*: 5 mg (low), 10 mg (medium), 23 mg (high).
- *Galantamine*: 8 mg (low), 16 mg (medium), 24 mg (high), 32–36 mg (very high).
- *Rivastigmine*: Oral (6 and 12 mg/day) or transdermal (4.6 mg (very low); 9.5 mg (low); 13.3 mg (moderate); 17.4 mg (high)).
- *Memantine*: Immediate-release (20 mg/day) or extended-release (28 mg/day).

### Outcomes

1. Categories of AEs like SAEs were defined according to the criteria reported in the individual trials to ensure consistency with the original study designs and to enable the inclusion of the widest range of eligible evidence. In line with the majority of studies, SAEs were considered as events requiring hospital admission, life-threatening outcomes, or death. This approach was chosen to address the heterogeneity in adverse event reporting, as some trials reported only aggregated SAE data without detailed classifications, while others applied different taxonomies. The same principle was applied to other categories of adverse events (e.g., gastrointestinal, central nervous system). If studies did not report aggregate categories of adverse events, they will be counted using the MedDRA System definitions. A similar approach was used by Battle et al. [24]. Primary Outcomes:
  *◦ Overall adverse events (AEs)*: we used the number of patients with any adverse events. If not available, we will use the number of adverse events. Usually, studies report AEs reported in ≥ 5% of participants or ≥ two individuals per study to avoid the number of events being higher than the total.
  *◦ Serious adverse events (SAEs)*: Defined as events requiring hospitalization, life-threatening outcomes (e.g., syncope, stroke, myocardial infarction), or death.
  *◦ Discontinuation rates due to AEs*.
2. Secondary Outcomes:
  ◦ When applicable, the following Specific AE categories will be explored:
    ▪ Gastrointestinal (e.g., nausea, diarrhea).
    ▪ Central nervous system (e.g., dizziness, headache).
    ▪ Psychological (e.g., agitation).
    ▪ Sleep disturbances.
    ▪ Falls.
    ▪ Skin-related adverse events.
    ▪ Cardiovascular adverse events.

### Data extraction and analysis plan

A standardized Excel sheet will be used for data extraction, capturing study characteristics, intervention details, and outcomes. Licensed cholinesterase inhibitors and memantine are available in a range of doses, and trials often include a dose-titration period, leading to the reporting of dose ranges (e.g., donepezil 5–10 mg) due to methodological differences. Some studies allow participants to retreat to lower doses during titration, while others require continuation at the higher dose or discontinuation. This variability introduces heterogeneity and fragments the data, resulting in clinically insignificant findings. To address this, we propose a methodology that consolidates dose ranges into the maximum intended therapeutic dose. For example, donepezil 5–10 mg would be considered equivalent to 10 mg, provided exploratory analyses show no significant subgroup differences between the dose ranges.

To validate this approach, an exploratory analysis will be conducted using pairwise meta-analyses and subgroup comparisons. For instance, we will compare outcomes such as adverse events (AEs) across dose ranges (e.g., donepezil 5 mg, 5–10 mg, and 10 mg) to test for significant subgroup differences. We hypothesize that no significant differences will exist between adjacent dose ranges, justifying the consolidation of 5–10 mg into 10 mg. This approach will be applied consistently across all drugs, including rivastigmine and galantamine, in line with existing literature. Transdermal patches will be treated as separate nodes and will not be condensed with oral drugs. While this method may overestimate or underestimate the risk associated with the higher dose of an interval, we will mitigate this limitation by reporting both the consolidated and original dose-range analyses as supplementary material. If significant differences emerge, they will be discussed in detail.

When applicable, stratification by dementia subtype (e.g., AD vs. non-AD), and AD disease severity (e.g., mild vs. moderate-severe AD based on MMSE or CDR scores) will be conducted. This approach addresses a significant problem in the literature by providing clinically actionable results, as dose ranges are not informative in clinical practice. Additionally, it allows for the identification of outliers and methodological inconsistencies, such as inflated AE rates due to flawed data collection. The exploratory nature of this analysis will also help identify gaps in the literature and provide new insights into the safety and efficacy of these drugs. By addressing the limitations of dose-titration trial designs, this innovative methodology aims to generate clinically relevant and methodologically sound evidence for the use of cholinesterase inhibitors and memantine in dementia care. Arms using non-standard dosing regimens (e.g., rivastigmine < 6 mg oral, donepezil < 5 mg) will be excluded from the analysis.

### Statistical analysis and quality assessment

All the statistics will be conducted using netmeta, dmetar, ggplot2, metafor, and gemtc packages on R statistical software version 4.4.1. We will perform a frequentist random-effects NMA using the graph-theoretical approach with appropriate handling of multi-arm trials (accounting for within-study correlations) so that each comparison is entered once per study. Dichotomous outcomes (AEs, SAEs, discontinuations) will be pooled as risk ratios (RRs) with 95% CIs. Between-study variance (τ^2^) will be estimated primarily by Restricted Maximum Likelihood (REML). Publication bias will be explored through inspection of the Doi plot with the LFK index. The closer to zero the LFK index is, the better the symmetry and the less risk of publication bias (-1 ≥ LFK index ≤ 1 will indicate symmetry).

We will present network graphs, league tables, and treatment rankings via SUCRA with 95%CI. Heterogeneity may result from changes in study design, inclusion/exclusion criteria, and trial procedures; hence, it will be graded as low, moderate, or high using the I2 test and significant or not by the Chi-square test (*P* < 0.1). Inconsistency will be examined via node-splitting and loop-specific inconsistency factors with 95% CIs. Suppose inconsistency is detected (*p* < 0.10 or clinically important magnitude). Beyond I^2^, we will report τ^2^ for the network and per outcome; use influence diagnostics (leave-one-out analyses) to identify influential studies; and conduct prespecified subgroup analyses to explain heterogeneity when applicable. A priori covariates are dementia subtype (AD vs non-AD; VaD; DLB/PDD), AD severity (mild vs moderate–severe by MMSE/CDR), and RoB2 overall judgement (low/some concerns/high).

### Quality assessment

The quality of included RCTs will be assessed using the Cochrane risk of bias tool (RoB2), which contains five domains to assess the overall quality of RCTs as either low, some concerns, or high risk of bias. The tool contains the following domains: (1) randomization process, (2) deviation from intended intervention, (3) missing outcome data, (4) measurement of outcomes, and (5) selection of the reported results [51]. Additionally, GRADE with a minimally contextualized approach will be used to assess the certainty of the evidence from the network meta-analysis [52]. Due to the large number of comparisons expected in our study, we will apply GRADE to the best and worst interventions in each outcome and statistically significant comparisons in the network. Certainty will start at high and be downgraded based on risk of bias, publication bias, Indirectness, inconsistency, and Imprecision, and upgraded for large effects, dose–response gradients, or residual confounding.

## Discussion

Our NMA results could significantly impact the global population, with over 55 million dementia cases worldwide and nearly 10 million new cases each year, contributing to an estimated global cost of $1.3 trillion [1–3]. In addition to reducing the rising caregiver’s psychological, mental, physical, and financial burdens [6–8]. Since effective dementia therapies are limited, ChEIs and memantine remain the principal medications for AD and non-AD dementias. Despite moderate benefits, ADRs, and high costs, they have been reported to reduce mortality, disability, and dependency [16–19, 21, 29, 33]. Previous NMAs have shown significant limitations, particularly in comparing different ChEI agents and memantine across dosages and formulations, focusing mainly on AD dementia and intervention efficacy, while overlooking other safety profiles, SAEs, and treatment withdrawals due to ADRs [24, 45–47]. This study aims to provide the most comprehensive and up-to-date comparative evaluation of the gold standard of evidence (RCTs) to determine the benefit-risk and cost-efficient profiles of different doses and formulations of ChEIs and memantine in the management of various forms of dementia. We intend to fill gaps in previous analyses to identify effective yet safe dementia treatment for evidence-based clinical decision-making and policy regulations while highlighting limitations to guide future research improvements. This study will have some limitations. Including only RCTs may not provide a comprehensive view of adverse events compared to long-term cohort studies. RCT sample sizes are usually insufficient to detect AEs occurring in < 1% of the population. However, our choice was made to provide estimates based on the highest quality of evidence. Our findings shall be correlated and augmented by the findings of long-term large observational and health-records studies.

## Supplementary Information

Supplementary Material 1.

## Data Availability

Data sharing is not applicable to this article as no datasets were generated or analyzed during the current study.

## Abbreviations

AD: Alzheimer’s Disease
AEs: Adverse Events
ChEIs: Cholinesterase Inhibitors
CI: Confidence Interval
CDR: Clinical Dementia Rating
CNS: Central Nervous System
DBL: Dementia with Lewy Bodies
DSM-5: Diagnostic and Statistical Manual of Mental Disorders, 5th Edition
FTD: Frontotemporal Dementia
ICD-11: International Classification of Diseases, 11th Revision
MMSE: Mini-Mental State Examination
NMA: Network Meta-Analysis
NMDAR: N-methyl-D-aspartate Receptor
PDD: Parkinson’s Disease Dementia
RCT: Randomized Controlled Trial
RoB2: Risk of Bias Tool 2
SAEs: Serious Adverse Events
SUCRA: Surface Under the Cumulative Ranking
TBI: Traumatic Brain Injury
VaD: Vascular Dementia
VCI: Vascular Cognitive Impairment
