# Supplementary material for: Comparative safety of cholinesterase inhibitors and memantine for dementia: a protocol for a network meta-analysis of randomized controlled trials
Source: Syst Rev. 2025 Nov 4;14:213. doi: 10.1186/s13643-025-02961-6 (PMC12584256; doi:10.1186/s13643-025-02961-6)
Supplement: Supplementary file 1 — Supplementary Material 1. [file 13643_2025_2961_MOESM1_ESM.docx]

**PRISMA-P (Preferred Reporting Items for Systematic review and Meta-Analysis Protocols) 2015 checklist: recommended items to address in a systematic review protocol***

| Section and topic | Item No | Checklist item |
| --- | --- | --- |
| ADMINISTRATIVE INFORMATION | | |
| Title: |  |  |
| Identification | 1a | \|  \| \| --- \|  \| p.1, L1–3 Titled as *“Comparative safety of cholinesterase inhibitors and memantine for dementia: A protocol for a network meta-analysis of randomized controlled trials”* \| \| --- \| |
| Update | 1b | NA |
| Registration | 2 | p.2, L94 Registered in PROSPERO: CRD4202564290 |
| Authors: |  |  |
| Contact | 3a | p.2, L22–28 |
| Contributions | 3b | p.18, L404–17 |
| Amendments | 4 | NA |
| Support: |  |  |
| Sources | 5a | p.18, L401 This study does not have external funding |
| Sponsor | 5b | p.18, L401 This study does not have external funding |
| Role of sponsor or funder | 5c | NA |
| INTRODUCTION | | |
| Rationale | 6 | p.6–9, L97–199 |
| Objectives | 7 | p.9, L200–210 |
| METHODS | | |
| Eligibility criteria | 8 | p.9–10, L216–242 |
| Information sources | 9 | p.9–10, L217–224 |
| Search strategy | 10 | p.9–10, L220–226 The complete search stragies for each database as following:  **PubMed**: (("Cholinesterase Inhibitors"[Mesh] OR "cholinesterase inhibitor*"[tiab] OR "acetylcholinesterase inhibitor*"[tiab] OR donepezil[tiab] OR galantamine[tiab] OR galanthamine[tiab] OR rivastigmine[tiab] OR memantine[tiab]) AND ("Dementia"[Mesh] OR dementia[tiab] OR "Alzheimer Disease"[Mesh] OR Alzheimer*[tiab] OR "vascular dementia"[tiab] OR "Lewy body dementia"[tiab] OR "dementia with Lewy bodies"[tiab] OR "Parkinson* disease dementia"[tiab] OR "frontotemporal dementia"[tiab])) AND ("Randomized Controlled Trial"[Publication Type] OR "Controlled Clinical Trial"[Publication Type] OR randomized[tiab] OR randomised[tiab] OR "random allocation"[tiab] OR "randomly assigned"[tiab] OR RCT[tiab])  **Web of Science:** TS=((acetylcholinesterase inhibitor* OR cholinesterase inhibitor* OR donepezil OR galantamine OR galanthamine OR rivastigmine OR memantine)) AND TS=((dementia OR Alzheimer OR "Alzheimer’s disease" OR "vascular dementia" OR "Lewy body dementia" OR "dementia with Lewy bodies" OR "Parkinson* disease dementia" OR "frontotemporal dementia")) AND TS=((randomized OR randomised OR "controlled trial" OR "controlled clinical trial" OR RCT OR "random allocation" OR "randomly assigned"))  **Cochrane library**: (acetylcholinesterase inhibitor* OR cholinesterase inhibitor* OR donepezil OR galantamine OR galanthamine OR rivastigmine OR memantine) AND (dementia OR Alzheimer OR "Alzheimer’s disease" OR "vascular dementia" OR "Lewy body dementia" OR "dementia with Lewy bodies" OR "Parkinson* disease dementia" OR "frontotemporal dementia") AND (randomized OR randomised OR "controlled trial" OR RCT OR "clinical trial" OR "random allocation" OR "randomly assigned")  **Scopus**: TITLE-ABS-KEY((acetylcholinesterase inhibitor* OR cholinesterase inhibitor* OR donepezil OR galantamine OR galanthamine OR rivastigmine OR memantine)) AND TITLE-ABS-KEY((dementia OR Alzheimer OR "Alzheimer’s disease" OR "vascular dementia" OR "Lewy body dementia" OR "dementia with Lewy bodies" OR "Parkinson* disease dementia" OR "frontotemporal dementia")) AND TITLE-ABS-KEY((randomized OR randomised OR "controlled trial" OR "controlled clinical trial" OR RCT OR "random allocation" OR "randomly assigned")) |
| Study records: |  |  |
| Data management | 11a | p.10, L227–229 |
| Selection process | 11b | p.10, L227–233 |
| Data collection process | 11c | p.11, L279–281 |
| Data items | 12 | p.10–13, L243–250; L279–285 |
| Outcomes and prioritization | 13 | p.11–12, L251–278 |
| Risk of bias in individual studies | 14 | p.14, L334–339 |
| Data synthesis | 15a | p.12–14, L312–327 |
|  | 15b | p.12–13, L317–319 |
|  | 15c | p.13–14, L327–331 |
|  | 15d | p.12–14, L323–325 |
| Meta-bias(es) | 16 | p.14, L319–321 |
| Confidence in cumulative evidence | 17 | p.14–15, L339–345 |

*** It is strongly recommended that this checklist be read in conjunction with the PRISMA-P Explanation and Elaboration (cite when available) for important clarification on the items. Amendments to a review protocol should be tracked and dated. The copyright for PRISMA-P (including checklist) is held by the PRISMA-P Group and is distributed under a Creative Commons Attribution Licence 4.0.**

*From: Shamseer L, Moher D, Clarke M, Ghersi D, Liberati A, Petticrew M, Shekelle P, Stewart L, PRISMA-P Group. Preferred reporting items for systematic review and meta-analysis protocols (PRISMA-P) 2015: elaboration and explanation. BMJ. 2015 Jan 2;349(jan02 1):g7647.*
